# Prediction of the next highly pathogenic avian influenza pandemic that can cause illness in humans

**DOI:** 10.1186/s40249-015-0083-8

**Published:** 2015-11-27

**Authors:** Zhong-Wei Zhang, Ting Liu, Jian Zeng, Yang-Er Chen, Ming Yuan, Da-Wei Zhang, Feng Zhu, Shu Yuan

**Affiliations:** College of Resources Science and Technology, Sichuan Agricultural University, Chengdu, 611130 China; Sichuan Kelun Pharmaceutical Comp. Ltd., Chengdu, 610072 China; Bioinformatic Study Centre, College of Life Sciences, Sichuan Agricultural University, Ya’an, 625014 China; Department of Plant Physiology, Umeå University, SE-901 87 Umeå, Sweden; Boyce Thompson Institute, Cornell University, Ithaca, NY 14850 USA; College of Life Sciences, Sichuan University, Chengdu, 610064 China; Department of Genetics, Development and Cell Biology, Iowa State University, Ames, IA 50011 USA; College of Horticulture and Plant Protection, Yangzhou University, Yangzhou, 225009 China

**Keywords:** Avian influenza viruses, Hemagglutinin, Point mutation, Translation efficiency, 5′-untranslated region

## Abstract

**Background:**

In recent years, avian influenza viruses (AIVs) have seriously threatened human health. Questions such as: why do AIVs infect humans?, how quickly can an AIV become pandemic?, and which virus is the most dangerous? cannot be sufficiently answered using current bioinformatic studies.

**Method:**

Secondary structures and energies of representative 5′-untranslated region (UTR) of the *HA* gene were calculated. Then their secondary structures and energies were re-calculated after one or two nucleotide substitutions were introduced into the *HA* 5′-UTR. Phylogenetic trees on the basis of hemagglutinin (HA) and polymerase basic protein 2 (PB2) amino acid sequences and *HA* 5′-UTR nucleotide sequences were constructed. The connection between the energy and translation efficiency of 5′-UTR was confirmed by *in vitro* coupled transcription/translation assay.

**Results:**

The simplicity of the secondary structure of the 5′-UTR of the *HA* gene determines the overall virus replication rate and transmission potential. Point mutation assays show that the 5′-UTR sequences of the *HA* gene in the influenza subtypes H2N2, H3N2, and H7N9 have greater variation potentials than other virus subtypes.

**Conclusion:**

Some high-virulent strains of avian influenza might emerge in the next two to three years. The H2N2 subtype, once disappeared in humans, may stage a comeback. The current outbreak of H7N9 may become pandemic and cause even more deaths, if one or two bases are substituted in the 5′-UTR sequence of the *HA* gene.

**Electronic supplementary material:**

The online version of this article (doi:10.1186/s40249-015-0083-8) contains supplementary material, which is available to authorized users.

## Multilingual abstracts

Please see Additional file 1 for translations of the abstract into the six official working languages of the United Nations.

## Background

During the past decade, an endless stream of avian influenza viruses (AIVs), including H1N1, H5N1, and H7N9, has emerged, leading to humans developing malignant respiratory diseases. The variation trend of influenza viruses needs to be urgently studied. In 1918, the Spanish flu, which was later confirmed as the H1N1 influenza virus, caused at least 20 million deaths worldwide (incomplete statistic) [1, 2]. Why was the Spanish flu so dangerous, and is there a possibility that there could be another outbreak like this in the future? These questions cannot be sufficiently answered using the current studies. In this paper, we attempt to answer these questions using new bioinformatic systems.

The hemagglutinin (HA) protein of the influenza subtypes that can infect humans preferentially recognizes α-2,6-linked sialic acids (SAs) (humanlike receptors), whereas the HA of avian influenza subtypes preferentially recognizes α-2,3-linked SAs (avian-like receptors). The alanine residue at position 160 (160A) in the H1N1 or H5N1 HA leads to an absence of glycosylation at positions 158 to 160 in HA, and permits virus binding to α-2,6-linked SAs [3–6]. Alternatively, a Leu → Gln mutation at position 226 (226L) in H7N9 HA changes the HA protein structure to exhibit a dual receptor-binding property [7, 8]. Either 160A in H1N1/H5N1 HA or 226L in H7N7/H7N9 (may also include H5N1) HA [9] may result in an influenza virus that can infect humans (mammals); N186K [5], G186V [5], or G228S [5, 7] in the HA protein structure may do the same thing.

Amino acid substitutions in basic polymerase (PB) proteins have been shown to be major determinants of a host’s range and transmission [10]. Whereas avian viruses, in principle, replicate at temperatures of around 41 °C (the temperature of the intestinal tract of birds), for replication in humans, viruses need to adapt to 33 °C (the temperature of the human upper respiratory tract, URT). The amino acid substitution Glu627 → Lys627 (E627K) in the polymerase complex protein PB2 has been associated with increased virus replication in mammalian cells at lower temperatures [11–14]. Amino acid substitutions D701N or S590G/R591Q in PB2 yield a similar phenotype to E627K [15]. However, the relationship between mutations of D701N/S590G/R591Q and their virulence is not clear for naturally occurring AIVs.

The above-mentioned genetically modified H5N1 or H5N1 hybrid viruses carrying 2009/H1N1 virus genes may cause airborne transmission. However, the transmission capability and virulence of artificial AIVs made in laboratories have been recorded as very low [5, 6, 12, 13]. We still do not know how far and how long a naturally occurring AIV would have to last to become pandemic. Previous phylogenetic analyses have suggested the origins and diversities of viruses [2, 14], however, these data were not generally related to the pathogenicity/transmissibility of AIVs. We still do not know which virus is the most dangerous.

In this paper, we introduce a new idea of a third prerequisite to airborne transmission: the simplicity of the secondary structure of the 5′-untranslated region (UTR) sequence of the *HA* gene, which may determine the overall virus replication and transmission rates. Through comprehensive analysis, we deduced that H2N2 might re-emerge, and that the current outbreak of H7N9 may become pandemic and cause even more deaths.

## Methods

### Retrieval and alignment of sequences

All sequences were acquired from the National Center for Biotechnology Information (http://www.ncbi.nlm.nih.gov). One hundred 5′-UTR sequences of representative H1N1, H2N2, H3N2, H5N1, H7N7, H7N9, H9N2, and H10N8 *HA* mRNAs were collected. Sampling criteria for each subtype— viruses from the three major hosts (avian, swine, and human influenza), and one or two other hosts—were collected if available (the majority of sequences were from the human host). Sequences of the viruses from different decades and different continents were collected. Some of the 5′-UTR sequences in the database were shorter than 43 bp, and so they were filled up to 40 bp. This was done by comparing them with homologous sequences from the same virus subtype, and then doing DNA sequence alignment using ClustalX 2.1 (http://www.clustal.org/) [16]. Alignment to 20 representative amino acid sequences (see Additional file 2: Table S1) was performed using ClustalX 2.1 [16].

### Prediction of 5′-UTR secondary structure

Secondary structures and energies of 60 5′-UTR sequences of *HA* mRNAs of AIVs (see Additional file 2: Table S2) were calculated using the latest version of RNADraw 1.1b2 (updated on June 18, 2012; http://www.rnadraw.com) [17], which can calculate structure energies of RNA structures with dangling ends. While running RNADraw, the temperature was set at 33 °C, taking into consideration that a relatively minor change in temperature (32–39 °C) would lead to a perturbation in the RNA structure [18]. When running the software, the option “use dangling end energies” was unchecked, but the “structure is part of multiloop” option was checked. The secondary structures and energies of representative 5′-UTR sequences were re-calculated after one or two nucleotide substitutions (both transversions and transitions were introduced).

### *In vitro* transcription/translation coupled reactions

Seven representative 40 bp 5′-UTR sequences (including two nucleotide substitutions) were introduced between the T7 promoter and the Luciferase template. Then, the *in vitro* transcription/translation coupled reaction was performed using the TNT® T7 Coupled Reticulocyte Lysate System (Promega BioSystems Sunnyvale Inc., Sunnyvale, CA, USA). After incubating at 33 °C for eight hours, luciferase activity was monitored using a GloMax® 20/20 Luminometer, following the manufacturer’s instructions (Promega) [19]. All assays were repeated three times, and the typical results shown with standard deviations (± SD). The Student’s *t*-test was performed for all data. *P*-values of 0.05 were considered significant.

### Phylogenetic analyses of HA and PB2 5′-UTR sequences

Hemagglutinin and polymerase basic protein 2 (PB2) amino acid sequences and 5′-UTR *HA* nucleotide sequences were aligned using ClustalX 2.1 [16]. Gaps resulting from the alignment were treated as missing data. Each phylogenetic tree was constructed using MEGA 6.0.5 (www.megasoftware.net), and employing the maximum-likelihood estimation, the JTT model, and 1,000 bootstrap replications [20].

## Results

### Mutations in HA and PB2 proteins

**Fig. 1:**
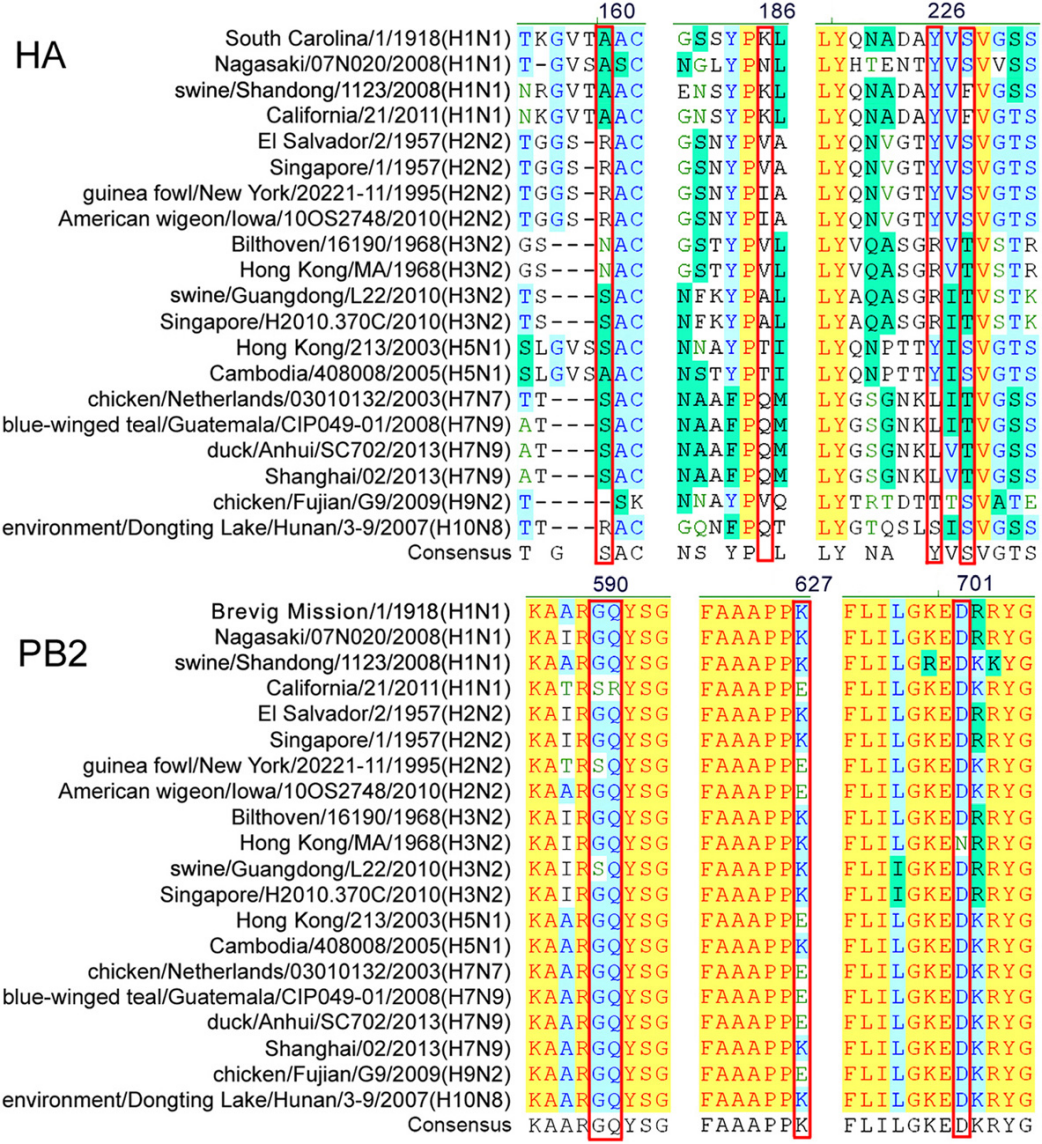
Alignment of HA and PB2 amino acid sequences from 20 representative AIVs (important mutation sites are marked with red boxes)

Hemagglutinin and PB2 protein sequences from 20 representative viruses (including the 1918 H1N1; see Additional file 2: Table S1) were aligned (see Fig. 1). Both the HA-160A and PB2-627K mutations were observed in the 1918 H1N1 subtype, thus possibly explaining its widespread dissemination. Both these mutations were also observed in the Nagasaki/07 N020/2008(H1N1) and swine/Shandong/1123/2008(H1N1) subtypes, thus explaining the high mortality rate of the 2008–2009 H1N1 flu pandemic, which killed around one in 10 people [21]. The HA-160A and PB2-627K mutations were also observed in the Cambodia/408008/2005(H5N1) subtype, thus the virus could lead to limited human-to-human transmission, and become much more dangerous than the Hong-Kong/213/2003(H5N1) subtype, which has neither of these two mutations. The PB2-627K mutation was not observed in the less-virulent strain California/21/2011(H1N1). Neither the HA-160A nor the HA-226L mutations were observed in the 1957 Asian flu (H2N2) or the 1968 Hong Kong flu (H3N2) subtypes, but the HA-186V and the PB2-627K mutations were observed, making them high-virulent strains [2]. The current outbreak of seasonal H3N2 can also infect humans [2], however neither the 160A, 186 V, nor 226L mutations have been observed in this subtype’s HA proteins, leading us to deduce that HA proteins may bind human receptors independent of these mutations. It is noteworthy that the HA-226L mutation has been observed in the majority of H7-type influenzas (see Fig. 1) [7, 8, 14], and therefore they might potentially infect humans. The HA-226L and PB2-627K mutations have both been observed in the Shanghai/02/2013(H7N9) subtype, thus leading to limited human-to-human transmissions of some H7N9 virus strains [7]. Contrastingly, the PB2-627K mutation has not been observed in the 2003 H7N7 subtype, thus only a few cases of human infections have been reported [22]. The HA-186V but not the PB2-627K mutation has been observed in the H9N2 subtype, while PB2-627K but not the HA-186V mutation has been observed in the H10N8 subtype. This means the potential of these strains to infect humans is relatively low.

### Secondary structure analysis of the 5′-UTR sequence of the *HA* gene

The third requirement for efficient human-to-human transmission is for sufficient virus particles to be carried in the respiratory droplet. For example, H5N1 is usually replicated in the lower region of the human respiratory tract, where the avian virus receptor is prevalent [23]. Meanwhile, seasonal influenza virus replicates in the upper region of the respiratory tract, and could be easily spread by sneezing and coughing [23].

Thus, AIVs need other factors to get sufficient virus particles in the respiratory droplet. Generally, approximately 500 HA and 100 neuraminidase (NA) spikes are distributed on the flu virus particle surface [24], making HA one of the most abundant proteins of the virus particle. Its synthesis may be the rate-limiting step of the whole virus particle assembly. There are many other proteins regulating the virus replication rate, such as non-structural protein 1 and the nucleic export protein [25]. However, they might be less important than HA in determining the overall virus replication rate.

A previous study suggested that a single nucleotide substitution in the 5′-UTR of a RNA molecule, where the elimination of the mismatch stabilizes the initiator helix by −3.6 kcal/mol, would lead to a 500-time decrease in its translation rate [26]. Therefore, we presume that the structure of the 5′-UTR of *HA* plus-strand RNA determines the HA protein translation efficiency [27], as well as the whole virus replication efficiency.

**Fig. 2:**
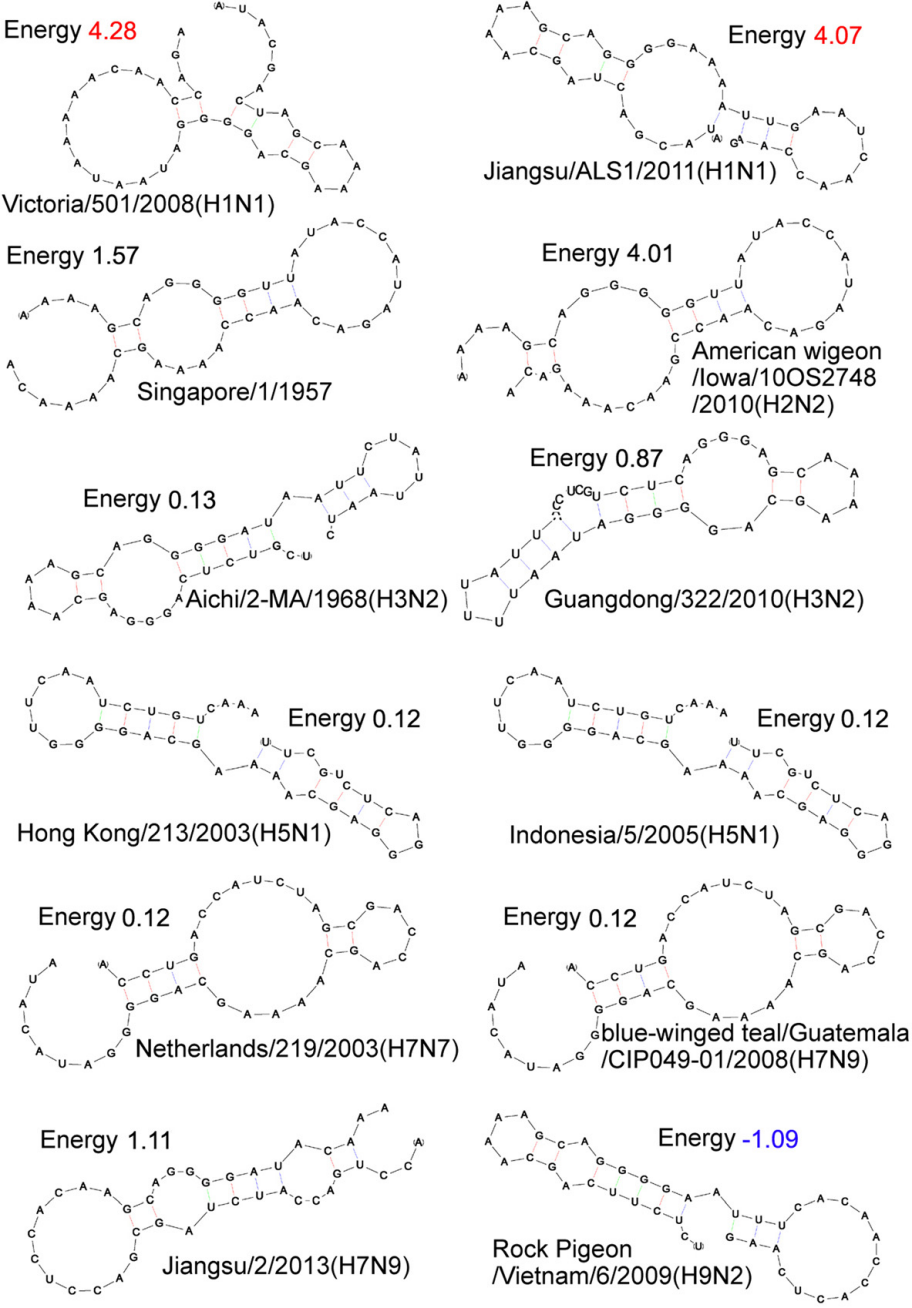
Secondary structures and energies of *HA* 5′-UTR from 12 representative AIVs (energy unit is kcal/mol)

One hundred representative *HA* 5′-UTR sequences from avian, swine, and human hosts were collected, and their secondary structures and energies were analyzed (see Fig. 2 and Additional file 2: Table S2). Most 5′-UTR sequences of H1N1 viruses have high energies (3.7–5.0 kcal), which means their secondary structures are relatively simple (less hairpins or branches). Correspondingly, the seasonal H1N1 subtype is the most pandemic influenza with the largest potential for transmission to humans [28]. The *HA* 5′-UTR of the 1957 H2N2 subtype has a slightly lower structure energy (1.3–1.6 kcal), but the *HA* 5′-UTR of the current H2N2 subtype has an energy as high as the H1N1. The human H3N2 subtype has a much lower energy (0.1–0.9 kcal), so it’s not as widespread as the H1N1 [28]. The energies of *HA* 5′-UTR sequences of most H5N1 subtypes are low (0.12 kcal). The H7N9 1988 and 2008 subtypes, and all types of H7N7 also have low energies (0.12 kcal). It is noteworthy that the 5′-UTR HA of the 2013 H7N9 subtype has a relatively higher energy of (1.11 kcal), although only three substitutions of bases occurred after 2008. Accordingly, a sudden outbreak of novel H7N9 influenza was reported in 2013 [7, 8]. The *HA* 5′-UTR sequences of H9N2 subtypes have the lowest energies, therefore, their translation efficiency should be relatively low. The 5′-UTR sequences of the H10N8 subtype *HA* mRNA in the current database are shorter than 20 bp, so they were not calculated.

### Variation potentials of HA 5′-UTR sequences

**Fig. 3:**
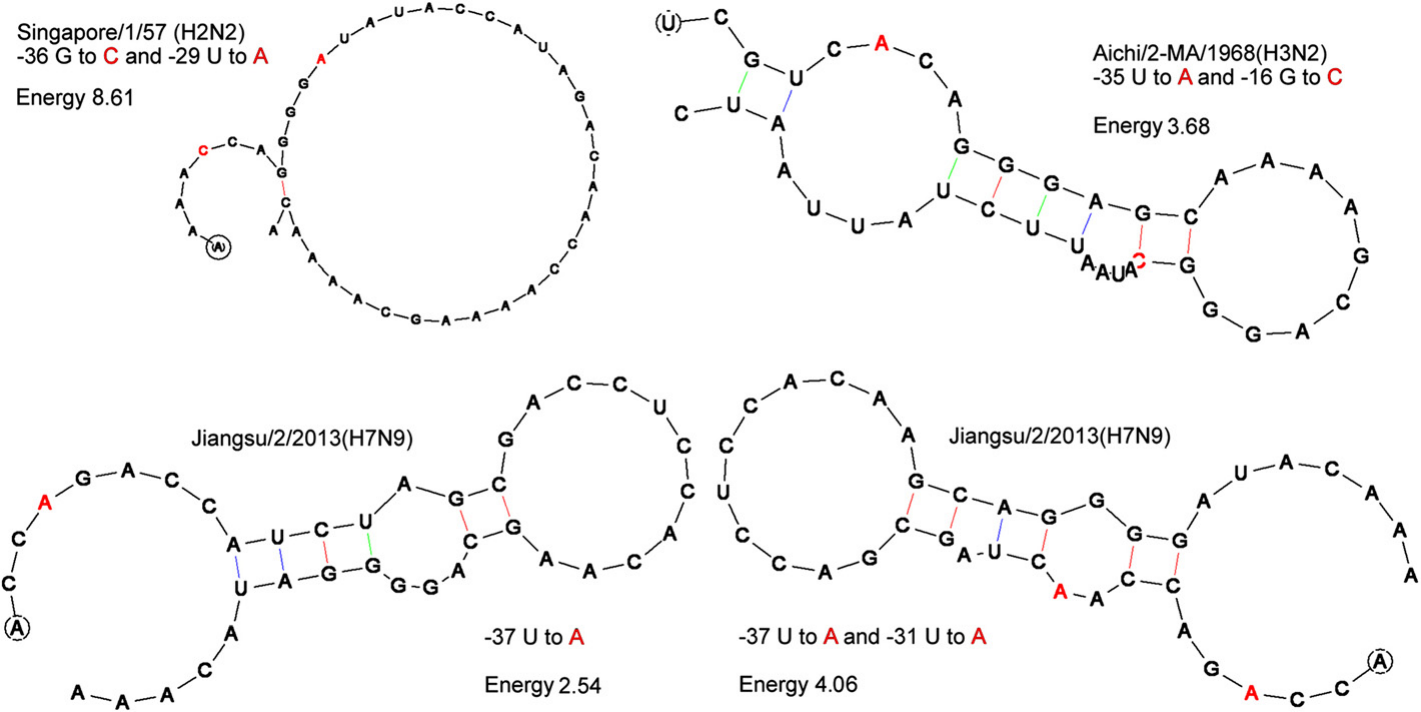
Secondary structures and energies of the *HA* 5′-UTR of the Singapore/1/57(H2N2), Aichi/2-MA/1968(H3N2), and Jiangsu/2/2013(H7N9) site-mutant variants (energy unit is kcal/mol)

The energies pertaining to 5′-UTR sequences of the 1957 H2N2 and 1968 H3N2 *HA* subtypes are much lower than those observed in the current outbreaks. They seem to be constantly slowly replicated. However, these sequences were not taken from patients, but from sub-inoculated materials (e.g., mouse lungs or egg-passages) [29]. The 5′-UTR may vary during these sub-inoculations. To know the potential variations of the *HA* 5′-UTR, point mutations were introduced into the sequences. For example, a -36G → C mutation in *HA* 5′-UTR of the Singapore/1/1957(H2N2) subtype increased the energy from 1.57 to 5.53 kcal; a -35C → G mutation resulted in an energy of 4.51 kcal; a -29U → A mutation resulted in an energy of 4.51 kcal; and the -36G → C and -29U → A double mutation resulted in the highest energy of 8.61 kcal (almost all hairpin or branch structures were eliminated; see Fig. 3). Regarding the Aichi/2-MA/1968(H3N2) subtype, a -37U → A mutation in *HA* 5′-UTR increased the energy from 0.13 to 3.02 kcal; a -35U → A mutation resulted in an energy of 3.44 kcal; a -16G → C mutation resulted in an energy of 3.2 kcal; and a -35U → A and -16G → C double mutation resulted in an energy of 3.68 kcal (see Fig. 3). Thus, the original structures of the *HA* 5′-UTR sequences of the 1957 H2N2 and 1968 H3N2 subtypes might be very simple (with high energies), therefore replicating very quickly and potentially killing thousands of people [2].

The *HA* 5′-UTR of the Indonesia/5/2005(H5N1) subtype can achieve a maximum energy level of 2.12 kcal (-8C → G or -7U → A), much lower than that of the H7N9 subtype. Human H5N1 infections largely declined after 2005. However, in the *HA* 5′-UTR of the Jiangsu/2/2013(H7N9) subtype, a -37U → A mutation increased the energy from 1.11 to 2.54 kcal; a -31U/-29U → A mutation increased it to 2.04 kcal; a -14G/-9G → C mutation increased it to 2.56 kcal; and the -37U → A and −31 → to A double mutation resulted in the highest energy of 4.06 kcal (see Fig. 3). Thus, the H7N9 subtype may become pandemic if mutations which make the structure more simple occur in the *HA* 5′-UTR.

For a pandemic outbreak, the H2N2 subtype needs a mutation in the PB2 coding region, while the H7N9 subtype needs a mutation in the HA non-coding region. Some selection pressure is usually applied on the coding region. The coding region varies less frequently than the non-coding region [30]. Thus, the H7N9 subtype is the most dangerous AIV.

### *In vitro* coupled transcription/translation assay with representative HA 5′-UTR sequences

**Fig. 4:**
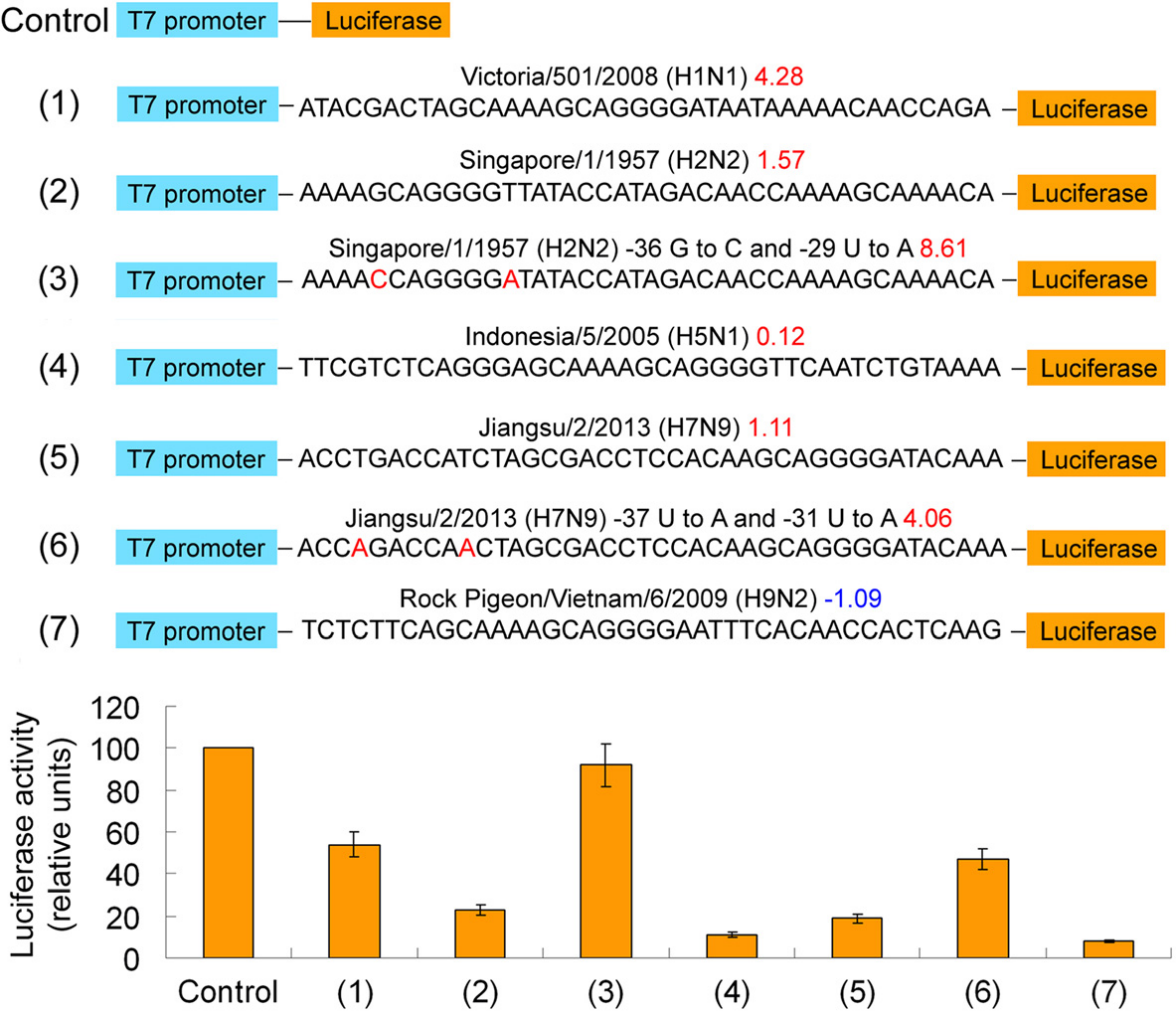
*In vitro* coupled transcription/translation assay with representative *HA* 5′-UTR sequences. Seven 40 bp 5′-UTR sequences (including two nucleotide substitution variants) were introduced between the T7 promoter and the Luciferase template, and then the *in vitro* transcription/translation coupled reaction was performed using the TNT® T7 Coupled Reticulocyte Lysate System. Luciferase activity of the control construct (T7 promoter – Luciferase fusion) was normalized at 100 relative units

Seven representative 40 bp 5′-UTR sequences were introduced between the T7 promoter and the Luciferase template, and then into the TNT® T7 Coupled Reticulocyte Lysate System. An *HA* 5′-UTR with a higher energy (simpler structure) resulted in a higher translation efficiency. For example, luciferase activity of the construct of the Singapore/1/1957(H2N2) subtype for the mutations -36G → C and -29U → A *HA* 5′-UTR (8.61 kcal) was almost 10 times of that of the Rock Pigeon/Vietnam/6/2009(H9N2) subtype *HA* 5′-UTR (−1.09 kcal) (see Fig. 4). *In vitro* coupled transcription/translation assay proved the above calculations.

### Phylogenetic analysis

Phylogenetic trees constructed on the basis of HA and PB2 proteins (see Additional file 2: Figure S1) and *HA* 5′-UTR (see Additional file 2: Figure S2) only show evolutionary cues without reflecting pathogenicity/transmissibility. They clustered mainly according to the species, but not around the key mutation sites or structure energies. The PB2 proteins didn’t cluster fully according to the species, indicating some possible reassortments (see Additional file 2: Figure S1). Some key mutations determine host type and viral replication efficiency, but cannot alter the phylogenetic tree, indicating that they may just be accidental events in AIV’s evolutionary history. In other words, pandemic and highly pathogenic AIVs may appear by chance. Regardless of whether a reassortment occurs, if the variation rate in the AIV pool is constant (mutations that happen in mammal hosts cannot be transferred back to the bird AIV pool), high-virulent strains of influenza should appear periodically independent of human infections. The coincidence of HA which can infect humans, human-replicable PB2, and highly energized *HA* 5′-UTR by certain mutations may happen after a certain time. The AIV pool size and its actual variation rate remain unknown, but based on other outbreaks (1918 H1N1, 1957 H2N2, 1968 H3N2, 2005 H5N1, 2009 H1N1, and 2013 H7N9), we can infer that the period might be about 40–50 years. Thus, one or two highly pathogenic strains might emerge in the next two to three years. This assumption aligns with the fact that all potential human infections and respiratory droplet transmissions are born within the viruses themselves, but not after they have already infected mammal hosts.

## Discussion

Influenza viruses of avian origins preferentially bind to α-2,3–linked SA receptors, whereas human influenza viruses recognize α-2,6–linked SA receptors. In humans, the α-2,6–linked SA receptors are predominantly present in the upper respiratory tract (URT), and the α-2,3–linked SA receptors are mainly present in the lower respiratory tract. In chickens and other birds, α-2,3–linked SAs dominate, yet both α-2,3–linked and α-2,6–linked SAs are present all over the respiratory and enteric tracts [3–6]. The differences in receptor distribution between humans and avian species are thought to determine the host restriction of influenza A viruses. Therefore, the switch in receptor specificity from avian α-2,3–linked SA to human α-2,6–linked SA is the most important requirement for efficient human-to-human transmission. Besides a switch in receptor specificity to facilitate infection of cells in the URT, increased virus production in the human URT and an efficient release of virus particles from the respiratory tract to yield airborne virus may also be required. Mutations in PB2 may help AIVs to be replicated efficiently at the same temperature as in the human URT [11–14]. In this paper, we suggest that the simplicity of the secondary structure of 5′-UTR of the *HA* gene determines its protein translation efficiency and the overall virus replication rate. We still do not know whether mutations in PB2 or highly energized *HA* 5′-UTR are more important for efficient replication in the human URT. Either might be enough for limited human-to-human transmission. So if the lower-temperature-activated PB2 and the highly energized *HA* 5′-UTR form at the same time, it may be a signal for a pandemic outbreak.

The highest energy of AIV *HA* 5′-UTR may theoretically reach 8.61 kcal, but energies of naturally occurring viruses are never greater than 5.0 kcal. A simplistic structure of *HA* 5′-UTR may result in excessive virus replication, leading to a quick death of the host (too soon to spread the virus), which is adverse to virus transmission. In addition, a balance between the properties endowed by HA and NA may be required to generate single particles. Imbalance of HA and NA tends to form virus aggregates [31].

There are similarities and differences between pathogenicity and transmissibility. The human immune system is familiar with seasonal flu viruses, such as H1N1 and H2N2, so the mortality rates of these strains are relatively low. On the contrary, unfamiliar viruses originating in poultry may cause a high mortality rate because the infection of an unfamiliar virus may set off a cytokine storm, resulting in a hyperimmune response to the virus and excessive cell death [32, 33]. Besides familiarity, another key factor determining pathogenicity is viral replication efficiency. Excessive viral replication may result in an excessive immune response, leading to a quick death. For example, the Netherlands/219/2003(H7N7) and the Hong Kong/470129/2013(H7N9) subtypes, two H7-type viruses, share a similarity of 96.8 %. However, a PB2-627K mutation as well as a much higher energized *HA* 5′-UTR have been observed in the latter (see Additional file 2: Table S2), meaning that the 2013 H7N9 subtype may be more virulent. The South Carolina/1/1918(H1N1) and the Nagasaki/07 N020/2008(H1N1) subtypes share a similarity of 88.9 %. The HA-160A and PB2-627K mutations have been observed in both these subtypes. However, the Nagasaki/07 N020/2008(H1N1) subtype is not as virulent and pandemic as the 1918 Spanish flu. One reason may be that the human immune system has become familiar with H1N1 viruses. Alternatively, the original 1918 H1N1 *HA* 5′-UTR may have an extraordinarily high energy (real sequence is not available).

## Conclusion

**Fig. 5:**
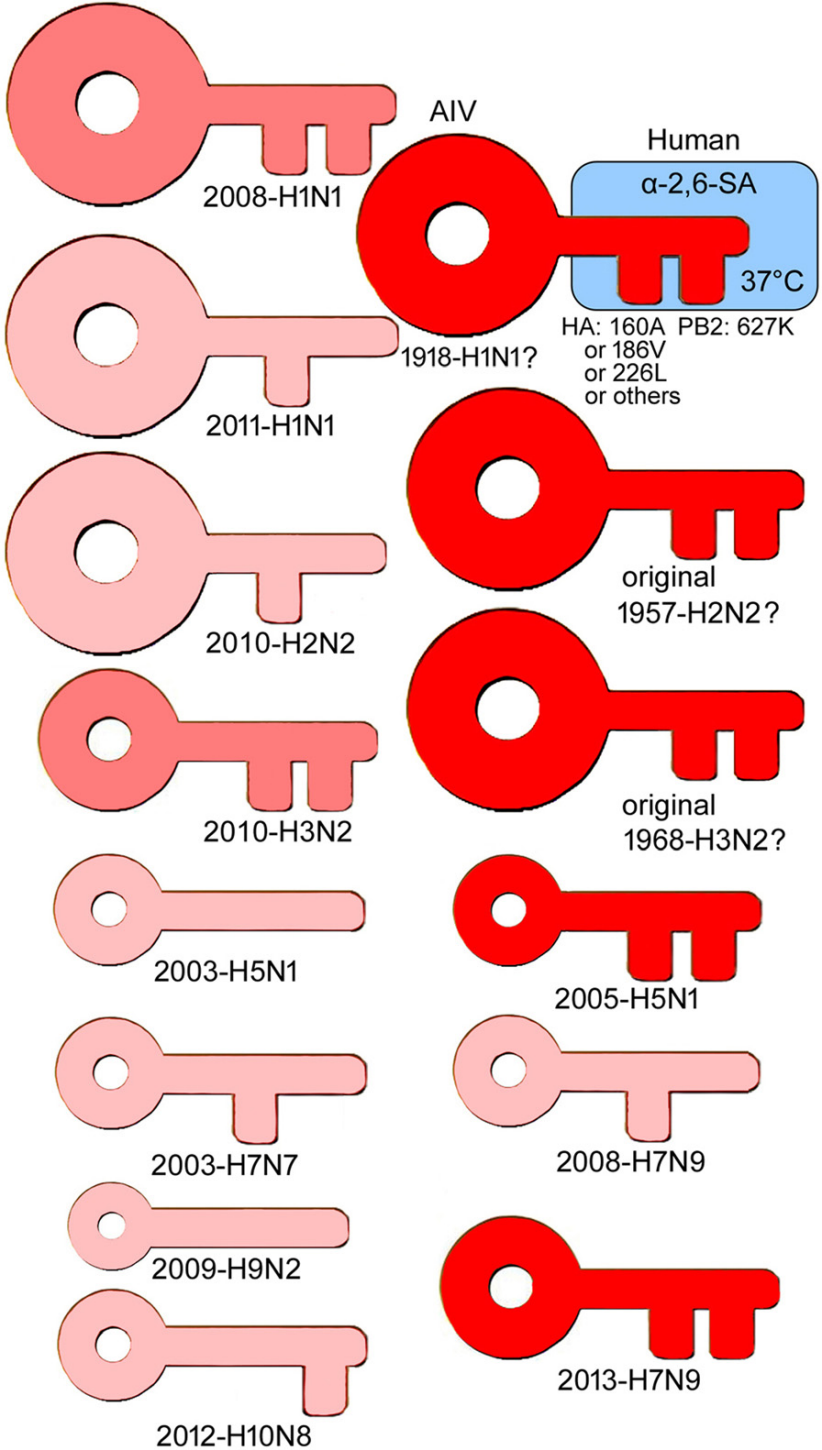
The analogy between lock and key and AIV pathogenicity and transmissibility. The human α-2,6-linked SA is like a lock and an AIV is like a key. The HA-160A/186V/226L (or other) and PB2-627K mutations are like the two teeth of the key. The *HA* 5′-UTR energy is like the handle of the key, the size of which determines the key’s twisting force. The depth of the red color shows the virulence level. Question marks denote that the original *HA* 5′-UTR sequences were not available

In summary, we can make the following analogy: the human α-2,6-linked SA is like a lock and an AIV is like a key. The HA-160A/226L and the PB2-627K mutations are like two teeth of the key. The energy of the *HA* 5′-UTR is like the handle of the key, the size of which determines the key’s twisting force (replication rate of the virus). Different AIVs have different teeth and different handle sizes. Viruses with both the HA-160A/226L and PB2-627K mutations and highly energized *HA* 5′-UTR may result in a pandemic (see Fig. 5).

## Additional files

Additional file 1:
**Multilingual abstracts in the six official working languages of the United Nations.** (PDF 273 kb) Additional file 2: Table S1. List of sequences used for the amino acid sequence alignment. **Table S2.** Sequences and energies of 5′-UTR of *HA* gene from 60 representative AIVs. **Figure S1.** Maximum-likelihood phylogenetic trees on the basis of HA and PB2 proteins. **Figure S2.** Maximum-likelihood phylogenetic trees on the basis of *HA* 5′-UTR sequences. (PDF 575 kb)

## Abbreviations

AIV: avian influenza virus
HA: hemagglutinin
NA: neuraminidase
PB: polymerase basic protein
SA: sialic acid
SD: standard deviation
URT: upper respiratory tract
5’UTR: 5’-untranslated region
