# Supplementary material for: Prediction of the next highly pathogenic avian influenza pandemic that can cause illness in humans
Source: Infect Dis Poverty. 2015 Nov 27;4:50. doi: 10.1186/s40249-015-0083-8 (PMC4661964; doi:10.1186/s40249-015-0083-8)
Supplement: Additional file 1: — Multilingual abstracts in the six official working languages of the United Nations. (PDF 273 kb) [file 40249_2015_83_MOESM1_ESM.pdf]

Translation of the abstract into the six official working languages of the United Nations

التنبؤ بالموجة الوبائية المقبلة من إنفلونزا الطيور وما يمكن أن تسببه من أمراض ووفيات لدى البشر

Zhong-Wei Zhang, Ting Liu, Jian Zeng, Yang-Er Chen, Ming Yuan, Da-Wei Zhang, Feng Zhu and Shu Yuan

ملخص
خلفية الموضوع: في السنوات الأخيرة، شكلت فيروسات أنفلونزا الطيور على اختلاف أنواعها (AIVs) تهديدًا خطيرًا على صحة الإنسان. واليوم تواجهنا العديد من الأسئلة: لماذا تصيب هذه النوعية من الفيروسات (AIVs) البشر؟، كيف تتحول إنفلونزا الطيور (AIV) بسرعة إلى وباء؟، وما هي أكثر الفيروسات المسببة لها خطورةً؟ لا يمكن الإجابة بشكل كاف على مثل هذه الأسئلة باستخدام المعلوماتية الحيوية الحالية أو ما يعرف بدراسات البيوإنفورماتيك فقط.
المنهج العلمي: تم حساب الهياكل الثانوية والطاقات الممثلة للتسلسل (5’-UTR) الخاص بالجين *HA*. ثم تمت إعادة حساب هذه الهياكل الثانوية والطاقات بعد إدماج واحدة أو اثنتين من عملية استبدال النوكليوتيدات إلى الجين (*HA* 5’-UTR). وهكذا تم تشييد أشجار تطور السلالات على الهيماغلوتينين القاعدي(HA) وبلمرة البروتين القاعدي 2 (PB2) مع تسلسل الأحماض الأمينية وتسلسل النوكليوتيدات (*HA* 5’-UTR). وقد تم التأكد من الاتصال بين كل من طاقة وكفاءة الترجمة لـ (5’-UTR) من قبل التناسخ / الترجمة للفحص ŭ Ṝ .
النتائج: إن بساطة الهيكل الثانوي لـ(5’-UTR) الخاص بجين *HA* يحدد بطريقة كلية معدل تكاثر الفيروس وقدرته على الانتقال. تظهر فحوصات موقع الطفرة أن تسلسل (5’-UTR) الخاص بجين *HA* في الأنواع الفرعية للأنفلونزا H2N2، H3N2، H7N9 له قدرة أكبر على الاختلاف والتغيير مقارنة بالأنواع الفرعية الأخرى من الفيروسات.
الخلاصة: بعض السلالات عالية الضراوة وشديدة العدوى من أنفلونزا الطيور قد تظهر في العامين أو الثلاثة أعوام القادمة. وبعد أن اختفت حالات الأنفلونزا من النوع الفرعي H2N2 في البشر، قد تكون على وشك الظهور من جديد. إن ما نشهده حاليًا من تفشي حالات الأنفلونزا من النوع الفرعي H7N9 قد يتحول إلى وباء ويسبب المزيد من الوفيات، إذا تعرض تسلسل "5‘-UTR" الخاص بجين "HA" لعملية استبدال لواحد أو اثنين من مكوناته القاعدية.

Translated from English version into Arabic by Muhammad Abdel Kader, through

TRANSLATORS
WITHOUT BORDERS

下一次人感染高致病性禽流感的预测

张中伟，刘婷，曾建，陈洋尔，袁明，张大伟，朱峰，袁澍

摘要
引言：近年来禽流感多次严重威胁到人类的健康。人们因此产生了一些疑问，比如为什么禽流感会感染人？下一次禽流感何时爆发？哪种禽流感病毒是最危险的？可惜的是，现有的研究结果还不足以回答这些问题。
方法：对病毒 RNA 的血凝素基因 *HA* 的 5'非翻译区（5’-UTR）的二级结构极其能量进行计算。对 *HA* 5’-UTR 进行一至两个点突变，再次计算其二级结构和能量大小。对 HA 蛋白、RNA 聚合酶亚基 2（PB2）和 *HA* 5’-UTR 的序列进行比对，构建聚类树。用体外转录-翻译偶联系统对上述 *HA* 5’-UTR 二级结构的计算结果进行验证。
结果：*HA* 5’-UTR 二级结构的复杂程度决定了病毒整体的复制速率和传播潜力。点突变的结果显示 H2N2、H3N2 和 H7N9 的变异潜力（再次爆发的可能性）高于其它病毒亚型。
结论：以上结果提示，在未来的 2-3 年内，可能再次爆发高致病力禽流感。曾经在人类社会消失的 H2N2 亚型有可能卷土重来。H7N9 的 *HA* 5’-UTR 只需要再发生一至两个点突变，就有可能获得更强的传播力，从而再次爆发。

Translated from English version into Chinese by Yuan Shu

Prédiction de la nouvelle pandémie de grippe aviaire hautement pathogène susceptible d’affecter les humains

Zhong-Wei Zhang, Ting Liu, Jian Zeng, Yang-Er Chen, Ming Yuan, Da-Wei Zhang, Feng Zhu and Shu Yuan

Résumé
Contexte: Plusieurs virus de la grippe aviaire ont gravement menacé la santé humaine ces dernières années. Les études bio-informatiques actuelles ne peuvent pas répondre suffisamment à des questions telles que : pourquoi ces virus infectent-ils les humains ? ou : quel virus est le plus dangereux ?
Méthode : Les structures secondaires et les énergies de régions non traduites (UTR) 5’ représentatives du gène *HA* ont été calculées. Leurs structures secondaires et énergies ont ensuite été recalculées après substitution d’un ou deux nucléotides dans l’UTR 5’ de *HA*. Des arbres phylogéniques ont été dressés sur la base des séquences d’acides aminés

de l'hémagglutinine (HA) et de la protéine de base de la polymerase 2 (PB2) et des séquences de nucléotides de l'UTR 5' de *HA* ont été construites. Le lien entre l'énergie et l'efficacité de traduction de l'UTR 5' a été confirmé par des essais de transcription/traduction couplés *in vitro*.
Résultats : La simplicité de la structure secondaire de l'UTR 5' du gène *HA* détermine la vitesse globale de réplication du virus et son potentiel de transmission. Les essais de mutation ponctuelle montrent que les séquences de l'UTR 5' du gène *HA* dans les sous-types de virus de la grippe H2N2, H3N2 et H7N9 présentent un plus fort potentiel de variation que d'autres sous-types de virus.
Conclusion : Des souches très virulentes de grippe aviaire pourraient surgir dans les deux ou trois ans qui viennent. Le sous-type H2N2, aujourd'hui disparu chez l'homme, pourrait faire son retour. L'épidémie actuelle de H7N9 pourrait devenir pandémique et causer encore plus de décès si une ou deux bases étaient substituées dans la séquences de l'UTR 5' du gène *HA*.

Translated from English version into French by Suzanne Assenat, through

Прогнозирование высокопатогенного птичьего гриппа, который может стать причиной человеческой пандемии

Чжун-Вей Чжан, Тин Лю, Цзянь Цзен, Ян-Эр Чэнь, Мин Юань, Да-Вэй Чжан, Фен Чжу и Шу Юань

Аннотация
История вопроса: В последние годы вирусы птичьего гриппа (ВПГ) серьезно угрожают здоровью человека. На вопросы: «Почему ВПГ заражают человека?», «Как быстро ВПГ может вызвать пандемию?» и «Какой вирус наиболее опасен?» нельзя ответить достоверно, используя только имеющиеся биоинформатические исследования.
Метод: Расчитаны вторичные структуры и силы репрезентативной 5'-нетранслируемой области (5'-НТО) HA-гена . Затем их вторичные структуры и силы были пересчитаны после одной или двух нуклеотидных замен в HA 5'-НТО. На основе геммаглютинина (HA) были построены филогенетические деревья, а также аминокислотные последовательности и нуклеотидные последовательности HA 5'-НТО на основе полимеразного белка 2 (PB2). Связь между энергией и эффективностью трансляции 5'-НТО была подтверждена транскрипцией/трансляцией in vitro.
Результаты: Простота вторичной структуры 5'-НТО HA гена обусловливает общую скорость репликации и потенциал передачи вируса. Анализ точечной мутации показал, что последовательности 5'-НТО HA гена в подтипах гриппа H2N2, H3N2 и H7N9 обладают большим потенциалом к изменениям по сравнению с другими подтипами вируса.
Заключение: В ближайшие 2-3 года могут появиться некоторые высоковирулентные штаммы птичьего гриппа. Подтип H2N2, некогда исчезнувший, может вернуться. Нынешняя вспышка H7N9 может привести к пандемии и унести еще больше жизней, если в последовательности HA 5'-НТО будет замещено одно или два основания.

Translated from English version into Russian by Jekaterina Merkuljeva, through

Predicción de la próxima pandemia de gripe aviaria, altamente patogénica, con posibilidades de producir enfermedad en el ser humano

Zhong-Wei Zhang, Ting Liu, Jian Zeng, Yang-Er Chen, Ming Yuan, Da-Wei Zhang, Feng Zhu y Shu Yuan

Resumen
Antecedentes: En los últimos años, los virus de la gripe aviaria han sido una seria amenaza para la salud humana. Preguntas como: ¿por qué los virus de la gripe aviaria afectan al ser humano? ¿qué tan rápido puede la gripe aviaria

pasar a ser pandémica? , y ¿qué virus son los más peligrosos? no pueden contestarse debidamente mediante el uso de estudios bioinformáticos.
Método: Se calcularon las estructuras y energías secundarias de regiones no traducidas 5’ representativas del gen *HA*. Posteriormente, sus estructuras y energías secundarias se re-calcularon luego de introducir sustituciones de uno o dos nucleótidos en las regiones no traducidas 5’ del HA. Se construyeron árboles filogenéticos con secuencias de aminoácidos y secuencias de regiones no traducidas 5’ del *HA* de hemaglutinina y la proteína básica polimerasa II. La conexión entre la energía y la eficiencia de la traducción de las regiones no traducidas 5’ fue confirmada por un ensayo *in vitro* en combinación con transcripción/traducción.
Resultados: La simplicidad de la estructura secundaria de las regiones no traducidas 5’ del gen HA determinan el índice general de replicación del virus y su posible transmisión. Los análisis de mutación puntual muestran que las secuencias no traducidas 5’ del gen HA en los subtipos H2N2, H3N2 y H7N9 de gripe tienen mayor potencial de variación que otros subtipos de virus.
Conclusión: Es posible que surjan cepas altamente virulentas de la gripe aviaria en los próximos dos a tres años. El subtipo H2N2, luego de su desaparición de los seres humanos, podría reaparecer. Es posible que el brote actual de H7N9 pase a ser pandémico y produzca incluso más muertes, si se sustituyen una o dos bases en la secuencia de las regiones no traducidas 5’ del gen HA.

Translated from English version into Spanish by Maria Alejandra Aguada, through

TRANSLATORS
WITHOUT BORDERS
