# Supplementary material for: Prediction of the next highly pathogenic avian influenza pandemic that can cause illness in humans
Source: Infect Dis Poverty. 2015 Nov 27;4:50. doi: 10.1186/s40249-015-0083-8 (PMC4661964; doi:10.1186/s40249-015-0083-8)
Supplement: Additional file 2: Table S1. — List of sequences used for the amino acid sequence alignment. Table S2. Sequences and energies of 5′-UTR of HA gene from 60 representative AIVs. Figure S1. Maximum-likelihood phylogenetic trees on the basis of HA and PB2 proteins. Figure S2. Maximum-likelihood phylogenetic trees on the basis of HA 5′-UTR sequences. (PDF 575 kb) [file 40249_2015_83_MOESM2_ESM.pdf]

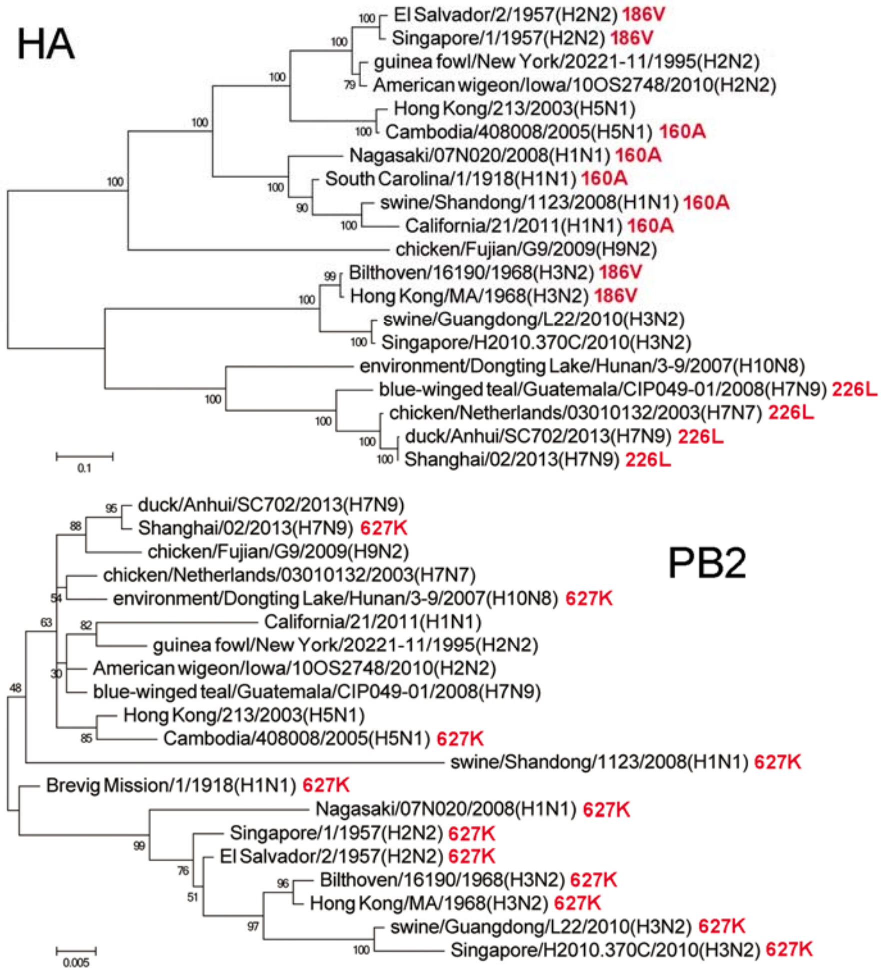

Figure S1 Maximum-likelihood phylogenetic trees of HA and PB2 proteins. Percent bootstrap values are presented for each clade. 160A, 186V and 627K mutations are marked with the red color after the species names.

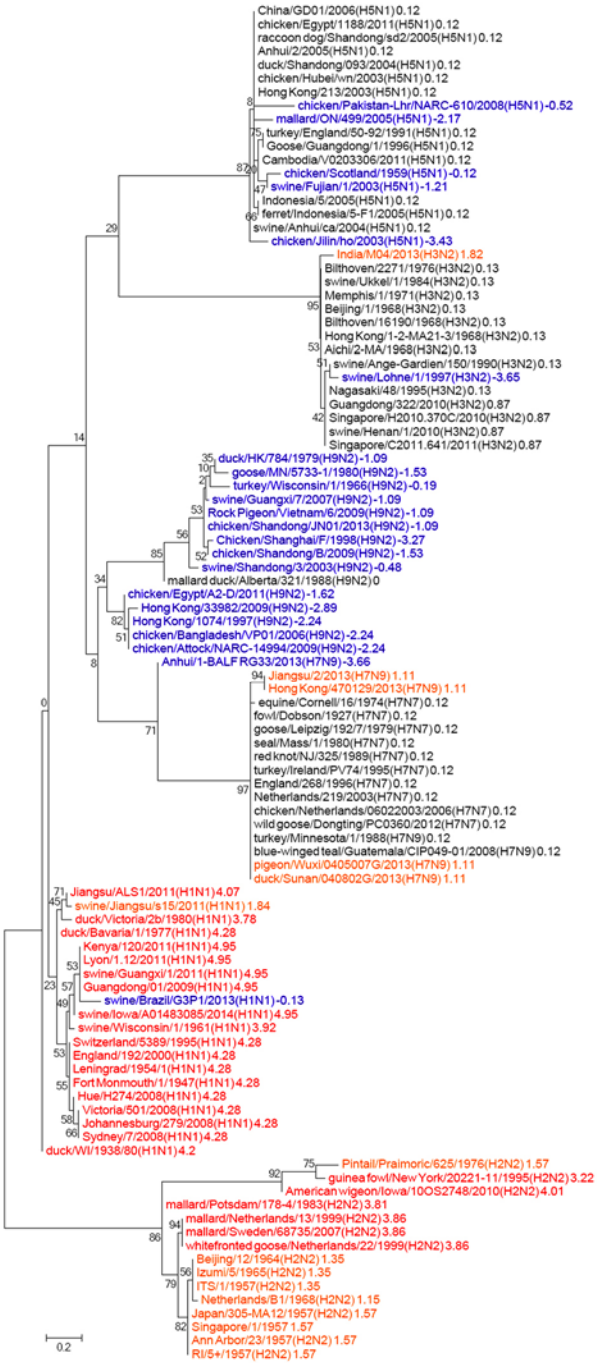

Figure S2 Maximum -likelihood phylogenetic trees of *HA* 5'-UTRs. Percent bootstrap values are presented for each clade. Energy values are shown after the species names. Red color means energy of the secondary structure > 3 kcal / mol. Orange color means energy values of 1-2 kcal / mol. Blue color means energy values of < 0 kcal / mol.

Table S1 List of sequences used for amino acid sequence alignment

Species name	*HA* gene<br>Accession number	*PB2* gene<br>Accession number
South Carolina/1/1918(H1N1) for *HA* gene	AF117241	
Brevig Mission/1/1918(H1N1) for *PB2* gene		DQ208309
Nagasaki/07N020/2008(H1N1)	CY043481	CY043478
swine/Shandong/1123/2008(H1N1)	GU646030	HM176665
California/21/2011(H1N1)	KC882293	KC882291
El Salvador/2/1957(H2N2)	HM204767	HM204774
Singapore/1/1957(H2N2)	FLASIN57HA	AY209935
guinea fowl/New York/20221-11/1995(H2N2)	CY014829	CY014836
American wigeon/Iowa/10OS2748/2010(H2N2)	CY133456	CY133463
Bilthoven/16190/1968(H3N2)	KC296480	KC296477
Hong Kong/MA/1968(H3N2)	HM641178	HM641145
swine/Guangdong/L22/2010(H3N2)	JX494706	JX494711
Singapore/H2010.370C/2010(H3N2)	JX437832	KF015095
Hong Kong/213/2003(H5N1)	AB212054	AB212051
Cambodia/408008/2005(H5N1)	AB598119	AB598126
chicken/Netherlands/03010132/2003(H7N7)	EF015551	EF015558
blue-winged teal/Guatemala/CIP049-01/2008(H7N9)	CY067670	CY067667
duck/Anhui/SC702/2013(H7N9)	CY147060	CY147057
Shanghai/02/2013(H7N9)	KF021597	KF021594
chicken/Fujian/G9/2009(H9N2)	JF715008	JN869514
environment/Dongting Lake/Hunan/3-9/2007(H10N8)	GQ325650	GQ325652

Table S2 Sequences and energies of *HA* 5'-UTR (40 bp) from 100 representative AIVs

Species name	Accession number	Energy (kcal)	Sequence
duck/WI/1938/80 (H1N1)	L25071	4.2	ATACGACTAGCAAAAGCAGGGGATA ATCAAATCAATCAAA
Fort Monmouth/1/1947 (H1N1)	U02085	4.28	ATACGACTAGCAAAAGCAGGGGAAA ATAAAAACAACCAAA
Leningrad/1954/1 (H1N1)	M38312	4.28	ATACGACTAGCAAAAGCAGGGGAAA ATAAAAACAACCAAA
swine/Wisconsin/1/1961 (H1N1)	AF091307	3.92	ATACGACTAGCAAAAGCAGGGGAAA ATAAAAGCAACCGAA
duck/Bavaria/1/1977 (H1N1)	AF091313	4.28	ATACGACTAGCAAAAGCAGGGGAAA ATTAAATCAACCAAA
duck/Victoria/2b/1980 (H1N1)	CY094927	3.78	ATACGACTAGCAAAAGCAGGGGGAA ATCAAATCAACCAAG
Switzerland/5389/1995 (H1N1)	AF386773	4.28	ATACGACTAGCAAAAGCAGGGGAAA ATAAAAACAACCAAA
England/192/2000 (H1N1)	CY125662	4.28	ATACGACTAGCAAAAGCAGGGGAAA ATAAAAACAACCAAA
Victoria/501/2008 (H1N1)	FJ743456	4.28	ATACGACTAGCAAAAGCAGGGGATA ATAAAAACAACCAGA
Johannesburg/279/2008 (H1N1)	FJ743452	4.28	ATACGACTAGCAAAAGCAGGGGATA ATAAAAACAACCAGA
Sydney/7/2008 (H1N1)	FJ743457	4.28	ATACGACTAGCAAAAGCAGGGGATA ATAAAAACAACCAGA
Hue/H274/2008 (H1N1)	CY104966	4.28	ATACGACTAGCAAAAGCAGGGGAAA ATAAAAACAACCAGA
Guangdong/01/2009 (H1N1)	HQ011408	4.95	ATACGACTAGCAAAAGCAGGGGAAA ACAAAAGCAACAAAA
Jiangsu/ALS1/2011 (H1N1)	HQ908440	4.07	ATACGACTAGCAAAAGCAGGGGAAA ATTGAATCAACCAAG
swine/Guangxi/1/2011 (H1N1)	JN222373	4.95	ATACGACTAGCAAAAGCAGGGGAAA ACAAAAGCAACAAAA
swine/Jiangsu/s15/2011 (H1N1)	JF820277	1.84	ATACGACTAGCAAAAGCAGGGGAAA ATTGAATCAATCAAG
Kenya/120/2011 (H1N1)	JQ396221	4.95	ATACGACTAGCAAAAGCAGGGGAAA ACAAAAGCAACAAAA
Lyon/1.12/2011 (H1N1)	KF897809	4.95	ATACGACTAGCAAAAGCAGGGGAAA ACAAAAGCAACAAAA
swine/Brazil/G3P1/2013 (H1N1)	KP027601	-0.13	ATACGACTTGCTTTAGCAGGGGAAA ACAAAAGCAACAAAA
swine/Iowa/A01483085/2014 (H1N1)	KP003901	4.95	ATACGACTAGCAAAAGCAGGGGAAA ATAAAAGCAACAAAA
ITS/1/1957 (H2N2)	CY125870	1.35	AAAAGCAGGGGTTATACCATAGACA ACCAAAAGCATAACA
Japan/305-MA12/1957 (H2N2)	CY087800	1.57	AAAAGCAGGGGTTATACCATAGACA ACCAAAAGCAAAACA
Singapore/1/1957 (H2N2)	L11142	1.57	AAAAGCAGGGGTTATACCATAGACA ACCAAAAGCAAAACA
Ann Arbor/23/1957 (H2N2)	CY125910	1.57	AAAAGCAGGGGTTATACCATAGACA ACCAAAAGCAAAACA
RI/5+/1957 (H2N2)	L20408	1.57	AAAAGCAGGGGTTATACCATAGACA ACCAAAAGCAAAACA
Beijing/12/1964 (H2N2)	KP098392	1.35	AAAAGCAGGGGTTATACCATAGACA ACCAAAAGCATAACA
Izumi/5/1965 (H2N2)	D13579	1.35	AAAAGCAGGGGTTATACCATAGACA ACCAAAAGCATAACA
Netherlands/B1/1968 (H2N2)	KM402809	1.15	AAAAGCAGGGGTTATACCATAGACA ACCAAAAGCGTAACA
Pintail/Praimoric/625/1976 (H2N2)	L11141	1.57	CAAAAGCAGGGGTTATACCATAGAC AACCAAAAGCAAACA
mallard/Potsdam/178-4/1983 (H2N2)	L11139	3.81	AAAAGCAGGGGTTATACCATAGACA ATCAAAGCAAAGACA
guinea fowl/New York/20221-11/1995 (H2N2)	CY014829	3.22	CAAAAGCAGGGGTTATACCATGAAC AACCAAACAAAGACA
whitefronted goose/Netherlands/22/1999 (H2N2)	KF695193	3.86	AAAAGCAGGGGTTATACCATAGACA ATCAAAAGCAGGACA
mallard/Netherlands/13/1999 (H2N2)	AY684893	3.86	AAAAGCAGGGGTTATACCATAGACA ATCAAAAGCAGGACA

mallard/Sweden/68735/2007 (H2N2)	CY121943	3.86	AAAAGCAGGGGTTATACCATAGACA ATCAAAAGCAGGACA
American wigeon/Iowa/10OS2748/2010 (H2N2)	CY133456	4.01	AAAAGCAGGGGGTTATACCATAGAC AACCGAACAAAGACA
Aichi/2-MA/1968 (H3N2)	KC895864	0.13	TCGTCTCAGGGAGCAAAAGCAGGGG ATAATTCTATTAATC
Hong Kong/1-2-MA21-3/1968 (H3N2)	CY080499	0.13	TCGTCTCAGGGAGCAAAAGCAGGGG ATAATTCTATTAATC
Bilthoven/16190/1968 (H3N2)	KC296480	0.13	TCGTCTCAGGGAGCAAAAGCAGGGG ATAATTCTATTAATC
Beijing/1/1968 (H3N2)	CY008156	0.13	TCGTCTCAGGGAGCAAAAGCAGGGG ATAATTCTATTAATC
Memphis/1/1971 (H3N2)	J02132	0.13	TCGTCTCAGGGAGCAAAAGCAGGGG ATAATTCTATTAATC
Bilthoven/2271/1976 (H3N2)	KC296467	0.13	TCGTCTCAGGGAGCAAAAGCAGGGG ATAATTCTATTAATC
swine/Ukkel/1/1984 (H3N2)	M73775	0.13	TCGTCTCAGGGAGCAAAAGCAGGGG ATAATTCTATTAATC
swine/Ange-Gardien/150/1990 (H3N2)	U07146	0.13	TCGTCTCAGGGAGCAAAAGCAGGGG ATAATTCTATCAACC
Nagasaki/48/1995 (H3N2)	AB019354	0.13	TCGTCTCAGGGAGCAAAAGCAGGGG ATAATTCTATTAACC
swine/Lohne/1/1997 (H3N2)	EF409249	-3.65	TCGTCTCAGGGAGCGAATGCAGGGG ATAATTCTATCAACC
Guangdong/322/2010 (H3N2)	CY091837	0.87	TCGTCTCAGGGAGCAAAAGCAGGGG ATAATTCTATTAACC
Singapore/H2010.370C/2010 (H3N2)	JX437832	0.87	TCGTCTCAGGGAGCAAAAGCAGGGG ATAATTCTATTAACC
swine/Henan/1/2010 (H3N2)	KF277766	0.87	TCGTCTCAGGGAGCAAAAGCAGGGG ATAATTCTATTAACC
Singapore/C2011.641/2011 (H3N2)	KF014149	0.87	TCGTCTCAGGGAGCAAAAGCAGGGG ATAATTCTATTAACC
India/M04/2013 (H3N2)	KP126930	1.82	TCGTCTCAGGGAGCTAAGGCAGGGG ATAAATCTATTAATC
chicken/Scotland/1959 (H5N1)	X07826	-0.12	TTCGTCTCAGGGAGCAAAAGCAGGG GTCTGATCTACTAAA
turkey/England/50-92/1991 (H5N1)	S68489	0.12	TTCGTCTCAGGGAGCAAAAGCAGGG GTATAATCTGTCAAA
Goose/Guangdong/1/1996 (H5N1)	AF144305	0.12	TTCGTCTCAGGGAGCAAAAGCAGGG GTATAATCTGTCAAA
swine/Fujian/1/2003 (H5N1)	AY747609	-1.21	TTCGTCTCAGGGAGCAAAAGCAGGG GTCTAATCTGCCAAA
chicken/Jilin/ho/2003 (H5N1)	DQ997355	-3.43	TTCGTCTCAGGGAGCGAAAGCAGGG GTCCAATCCGTCATG
Hong Kong/213/2003 (H5N1)	AB212054	0.12	TTCGTCTCAGGGAGCAAAAGCAGGG GTTCAATCTGTCAAA
chicken/Hubei/wn/2003 (H5N1)	DQ997147	0.12	TTCGTCTCAGGGAGCAAAAGCAGGG GTTCAATCTGTCAAA
duck/Shandong/093/2004 (H5N1)	AY854190	0.12	TTCGTCTCAGGGAGCAAAAGCAGGG GTTCAATCTGTCAAA
swine/Anhui/ca/2004 (H5N1)	DQ997392	0.12	TTCGTCTCAGGGAGCGAAAGCAGGG GTTCAATCTGTCAAA
Indonesia/5/2005 (H5N1)	CY116646	0.12	TTCGTCTCAGGGAGCAAAAGCAGGG GTTCAATCTGTAAAA
mallard/ON/499/2005 (H5N1)	FJ032001	-2.17	TTCGTCTCAGGGAGCAAAAGCAGGG GTCCAAACTATGAAA
Anhui/2/2005 (H5N1)	HM172072	0.12	TTCGTCTCAGGGAGCAAAAGCAGGG GTTCAATCTGTCAAA
ferret/Indonesia/5-F1/2005 (H5N1)	CY116654	0.12	TTCGTCTCAGGGAGCAAAAGCA GGGGTTCAATCTGTAAAA
raccoon dog/Shandong/sd2/2005 (H5N1)	EU420046	0.12	TTCGTCTCAGGGAGCAAAAGCAGGG GTTCAATCTGTCAAA
China/GD01/2006 (H5N1)	DQ835313	0.12	TTCGTCTCAGGGAGCAAAAGCAGGG GTTCAATCTGTCAAA
chicken/Pakistan-Lhr/NARC-610/2008 (H5N1)	JF827078	-0.52	CATTTCTCTTCGAGCAAAAGCAGGG GTTCAGTCTGTCAAA
Cambodia/V0203306/2011 (H5N1)	JN588805	0.12	TTCGTCTCAGGGAGCAAAAGCAGGG GTTTAATCTGTCAAA
chicken/Egypt/1188/2011 (H5N1)	JQ858479	0.12	TTCGTCTCAGGGAGCAAAAGCAGGG GTTCAATCTGTCAAA

fowl/Dobson/1927 (H7N7)	CY014992	0.12	ACCTGACCATCTAGCGACCAGCAAA AGCAGGGGATACAAA
equine/Cornell/16/1974 (H7N7)	EU375343	0.12	ACCTGACCATCTAGCGACCAGCAAA AGCAGGGGATACATA
goose/Leipzig/192/7/1979 (H7N7)	L43915	0.12	ACCTGACCATCTAGCGACCAGCAAA AGCAGGGGATACAAA
seal/Mass/1/1980 (H7N7)	K00429	0.12	ACCTGACCATCTAGCGACCAGCAAA AGCAGGGGATACAAA
red knot/NJ/325/1989 (H7N7)	CY005981	0.12	ACCTGACCATCTAGCGACCAGCAAA AGCAGGGGATACAAA
turkey/Ireland/PV74/1995 (H7N7)	AF028021	0.12	ACCTGACCATCTAGCGACCAGCAAA AGCAGGGGATACAAA
England/268/1996 (H7N7)	AF028020	0.12	ACCTGACCATCTAGCGACCAGCAAA AGCAGGGGATACAAA
Netherlands/219/2003 (H7N7)	AY338459	0.12	ACCTGACCATCTAGCGACCAGCAAA AGCAGGGGATACAAA
chicken/Netherlands/06022003/2006 (H7N7)	HE802065	0.12	ACCTGACCATCTAGCGACCAGCAAA AGCAGGGGATACAAA
wild goose/Dongting/PC0360/2012 (H7N7)	KC876683	0.12	ACCTGACCATCTAGCGACCAGCAAA AGCAGGGGATACAAA
turkey/Minnesota/1/1988 (H7N9)	CY014786	0.12	ACCTGACCATCTAGCGACCAGCAAA AGCAGGGGATACAAA
blue-winged teal/Guatemala/CIP049-01/2008 (H7N9)	CY067670	0.12	ACCTGACCATCTAGCGACCAGCAAA AGCAGGGGATACAAA
Jiangsu/2/2013 (H7N9)	KF226105	1.11	ACCTGACCATCTAGCGACCTCCACA AGCAGGGGATACAAA
Hong Kong/470129/2013 (H7N9)	KF952508	1.11	ACCTGACCATCTAGCGACCTCCACA AGCAGGGGATACAAA
pigeon/Wuxi/0405007G/2013 (H7N9)	KM879381	1.11	ACCTGACCATCTAGCGACCAGCAAA AGCAGGGGATACAAA
duck/Sunan/040802G/2013 (H7N9)	KM879373	1.11	ACCTGACCATCTAGCGACCAGCAAA AGCAGGGGATACAAA
turkey/Wisconsin/1/1966 (H9N2)	CY014663	-0.19	TCTCTTCAGCAAAAGCAGGGGAATA ACATAGCCAATCAAG
duck/HK/784/1979 (H9N2)	CY005632	-1.09	TCTCTTCAGCAAAAGCAGGGGAATT TCACAACCAACCAAG
goose/MN/5733-1/1980 (H9N2)	CY006042	-1.53	TCTCTTCAGCAAAAGCAGGGGAATT TCACAATTAACCAAA
mallard duck/Alberta/321/1988 (H9N2)	AY633276	0	TCTCTTCGAGCAAAGCAGGGGAATT TCACAATCAAACAAA
Hong Kong/1074/1997 (H9N2)	GU053179	-2.24	TCTCTTCGAGCAAAAGCAGGGGAAT TTCTCAACTAGCAAA
Chicken/Shanghai/F/1998 (H9N2)	AY743216	-3.27	TCTCTTCAGCAAAAGCAGGGGAATT TCACAACCAGTCAAA
swine/Shandong/3/2003 (H9N2)	GQ477289	-0.48	TCTCTTCGAGAAAAGCAGGGGTACT TCACAACCACTCAAG
chicken/Bangladesh/VP01/2006 (H9N2)	KC986294	-2.24	TCTCTTCGAGCAAAAGCAGGGGAAT TTCTCAACTAGCAAA
swine/Guangxi/7/2007 (H9N2)	CY075030	-1.09	TCTCTTCAGCAAAAGCAGGGGAATT TCACAACCATTCAAG
Rock Pigeon/Vietnam/6/2009 (H9N2)	AB753201	-1.09	TCTCTTCAGCAAAAGCAGGGGAATT TCACAACCACTCAAG
chicken/Shandong/B/2009 (H9N2)	JN683642	-1.53	TCTCTTCAGCAAAAGCAGGGGAATT TCACAACCACTCAAA
chicken/Attock/NARC-14994/2009 (H9N2)	JN540074	-2.24	TCTCTTCGAGCAAAAGCAGGGGAAT TTCTCAACTAGCAAA
Hong Kong/33982/2009 (H9N2)	KF188316	-2.89	TCTCTTCGAGCAAAAGCAGGGGAAT TTCTTAACTAGCAGA
chicken/Egypt/A2-D/2011 (H9N2)	KC017474	-1.62	TCTCTTCGAGCAAAAGCAGGGGAAT TTCACAACTAGCAAA
chicken/Shandong/JN01/2013 (H9N2)	KP006602	-1.09	TCTCTTCAGCAAAAGCAGGGGAATT TCACAACCACTCAAG
Anhui/1-BALF_RG33/2013 (H7N9)	CY187402	-3.66	TCTCTTCGAGCAAAAGCAGAGCGAA AGCAGGGGATACAAA
mallard/Sweden/7/2003 (H10N8) 20bp	CY060374		NNNNNNNNNNNNNNNNNNNNNNNNC AAAAGCAGGGGTCACA
duck/Guangdong/E1/2012 (H10N8) 20bp	JQ924786		NNNNNNNNNNNNNNNNNNNNNNAGC AAAAGCAGGGGTCACA
